# Voluntary Medical Societies in Kentucky

**Published:** 1837

**Authors:** 


### Art. V.—Voluntary Medical Societies in Kentucky.

We lately received, from a respectable correspondent at Princeton, Caldwell Co., Ky., a letter, from which we venture to make the following extract:—

"I observe you mention, in your travelling memoranda, the different Medical Societies lying in your route, last summer. If you had passed through our place, you would have discovered that we have a County Medical Society. It is about three years since ours was established; it still continues to go on regularly, and, I think, is of considerable advantage, both to our physicians and the people. There are no jealousies among our physicians, and they all move on with regularity, and the utmost friendship and harmony prevail amongst them. The annual meeting of our Society is on the second Saturday in May, and the quarterly meetings are on the same day in August, November and February; at each of which times, there is a paper read on some medical subject, and discussed by those who feel so disposed. The Society is called the Caldwell Medical Society. Dr. Throckmorton is President at this time, Dr. J. Webb Vice President, Dr. M. Goodwin Treasurer, Dr. McNary Corresponding Secretary, and Dr. William W. Throckmorton Recording Secretary. This is the first Medical Society established south of Green river, and we are endeavoring to have one established in every county in the State; or, at all events, in those south of Green river. If we find it impracticable to form a Society in each county, we shall endeavor to have District Societies; and if we succeed, we shall send delegates to meet at Frankfort, once a year, so as to regulate the practice of our State. I will just observe, that when our Society went into operation, our county contained eight or ten steam doctors; and one of them had a shop, and a strong support in Princeton; there were, besides, two others in town, who did not keep shops, but attended to other business, and kept their steam and medicines at their dwellings, and practiced when occasion offered. There is not now, and, for more than a year, has not been, a steam doctor in our town, and I do not believe there is one in the county."

We are happy to find a proper spirit among our friends in the Green River country. Voluntary association is the true principle of organization and action. Chartered societies are too often regarded as monopolies, and are exceedingly apt to become in bad odour with the people. In all the older counties of the Western States, there are physicians enough to form associations for mutual improvement, and why should they not do it? Are medical gentlemen raised, by their elementary education, above the necessity of seeking instruction from each other? Are they not, rather, of all the *castes* in society, the most in want of such opportunities? And this want is not confined to the young or the comparatively ignorant? Through every epoch of his professional life, the physician, however learned, needs the influence of professional association, to repress self-sufficiency, avert mannerism, and arouse his ambition—yes, to arouse his ambition! for, except when ambition suggests insulation as a means of accomplishing its ends, it languishes, without association; and, let the world say what it may, ambition, guided by justice and mercy, is the noblest virtue of a physician—one, indeed, without which, he is generally but a vapid and inert routinist. D.

